# Bleeding from jejunal varices formed at the Roux-en-Y jejunum site caused by the compression of the left renal vein after living donor liver transplantation with renoportal anastomosis

**DOI:** 10.1186/s40792-021-01129-3

**Published:** 2021-02-06

**Authors:** Wataru Nakanishi, Shigehito Miyagi, Kazuaki Tokodai, Atsushi Fujio, Toshiaki Kashiwadate, Kengo Sasaki, Yoshihiro Shono, Mineto Ohta, Yoshikatsu Saitoh, Michiaki Unno, Takashi Kamei

**Affiliations:** Department of Surgery, Tohoku University Graduate School of Medicine, 1-1 Seiryo-machi, Aoba-ku, Sendai, Miyagi Japan

**Keywords:** Liver transplantation, Nutcracker syndrome, Renoportal anastomosis, Variceal hemorrhage

## Abstract

**Background:**

Renoportal anastomosis is an option for the portal vein reconstruction of a liver transplantation with grade 4 portal vein thrombosis and a splenorenal shunt. Here, we report the case of gastrointestinal bleeding who underwent living donor liver transplantation (LDLT) with renoportal anastomosis.

**Case presentation:**

Six-year-old female patient who underwent LDLT with renoportal anastomosis at 1 year of age had severe anemia with normal liver function during the follow-up period. The varices at the Roux-en-Y jejunum were considered the source of bleeding, and the compression of the left renal vein, which is known as a cause of Nutcracker syndrome, seemed to induce venous hypertension through the splenorenal shunt, which might induce the formation of the varices. She underwent percutaneous transhepatic sclerotherapy of the varices, and the anemia improved at her last follow-up, 6 months after sclerotherapy. This is the first reported case of Roux-en-Y jejunal varices bleeding related to the compression of the left renal vein after LDLT was performed with renoportal anastomosis.

**Conclusions:**

Although renoportal anastomosis should be cautiously performed when there are no options for severe portal vein thrombosis, the status of the left renal vein and new collateral formation should be observed carefully during the follow-up period in pediatric cases of renoportal anastomosis.

## Background

Liver transplantation (LT) is the most curative therapy for end-stage liver disease. Portal vein thrombosis (PVT) is encountered in patients with liver cirrhosis and is recognized as an obstacle for LT due to technical problems and a high incidence of PVT recurrence after LT. Various surgical techniques such as portal vein (PV) venoplasty, interpositional grafts, and mesenteric jump grafts have been reported to perform LT in patients with PVT [1]. Renoportal anastomosis (RPA) is a portal reconstruction in which a recipient left renal vein (LRV) is anastomosed to a graft PV using an interpositional graft. RPA was first reported by Sheil et al. in 1997 [2], thereafter, RPA was performed in patients with PVT and splenorenal shunts (SRSs). However, there are few reports concerning pediatric cases and long-term results. We report the youngest case of a female pediatric LDLT performed with RPA [3]. During the patient’s follow-up period, gastrointestinal bleeding from varices of the Roux-en-Y (RY) jejunum occurred. Here, we report a rare case of RY varices caused by compression of the LRV in the patient in which RPA was performed.

## Case presentation

**Fig. 1:**
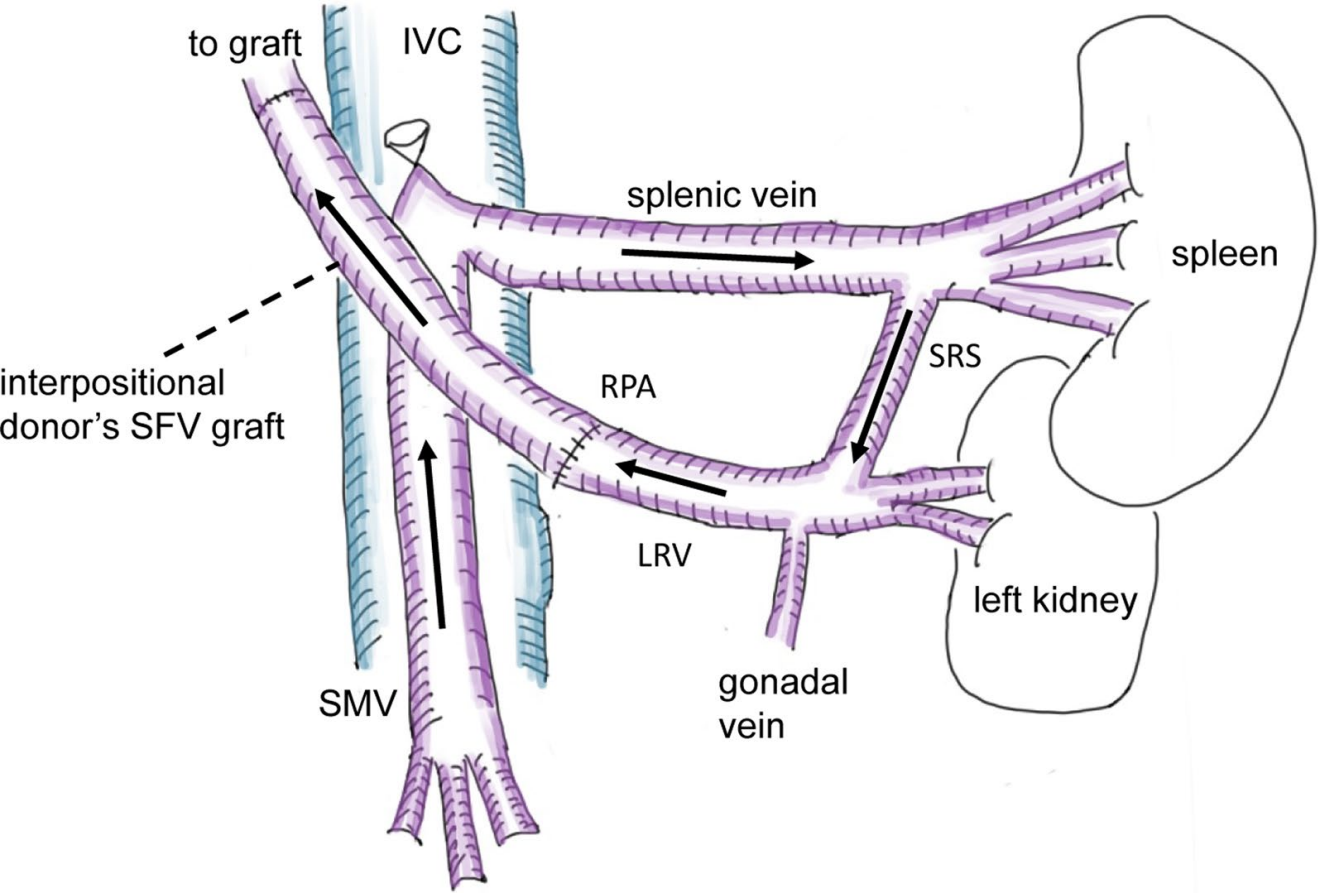
Schematic figure of RPA. The black arrows indicate blood flow from SMV to the liver graft. *IVC* inferior vena cava, *LRV* left renal vein, *RPA* renoportal anastomosis, *SFV* superficial femoral vein, *SMV* supramesenteric vein, *SRS* splenorenal shunt

A 1-year-old girl underwent LDLT in 2015 after a failed Kasai’s operation. The native PV trunk had become phlebosclerotic due to repetitive cholangitis, and a large SRS was developed. At the time of LDLT, PV reconstruction was performed by RPA using the donor’s superficial femoral vein graft as an interpositional graft (Fig. 1). The patient was treated using tacrolimus-based immunosuppression. After discharge, her operative course was uneventful, and she grew up normally until the age of six. During follow-period, Doppler ultrasonography has recorded consistently steady flow of intrahepatic portal vein.

**Fig. 2:**
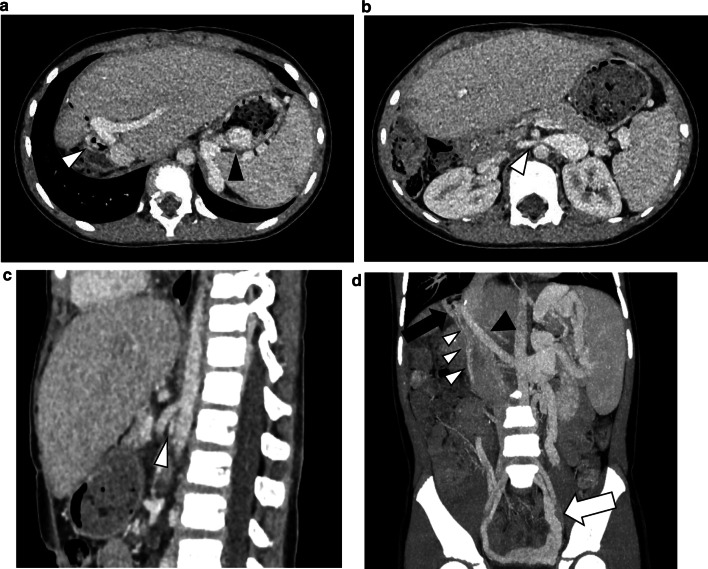
AB Axial CT image. **a** The white arrowhead shows the jejunal varices. The black arrowhead shows the gastric varices. **b** The white arrowhead indicates compression of the LRV between the SMA and the Ao. **c** Sagittal CT image. The white arrowhead indicates the narrow angle between the SMA and the Ao. **d** Multiplaner reconstruction CT image. The black arrow shows the varices of the RY jejunum. The white arrowheads indicate collaterals to the RY varices from the SMV. The black arrowhead indicates the patent RPA. Pelvic vein congestion (white arrow) is suggestive of increased venous pressure via the dilated gonadal vein. *Ao* abdominal aorta, *CT* computed tomography, *LRV* left renal vein, *RY* Roux-en-Y, *SMA* supramesenteric artery, *SMV* supramesenteric vein

**Fig. 3:**
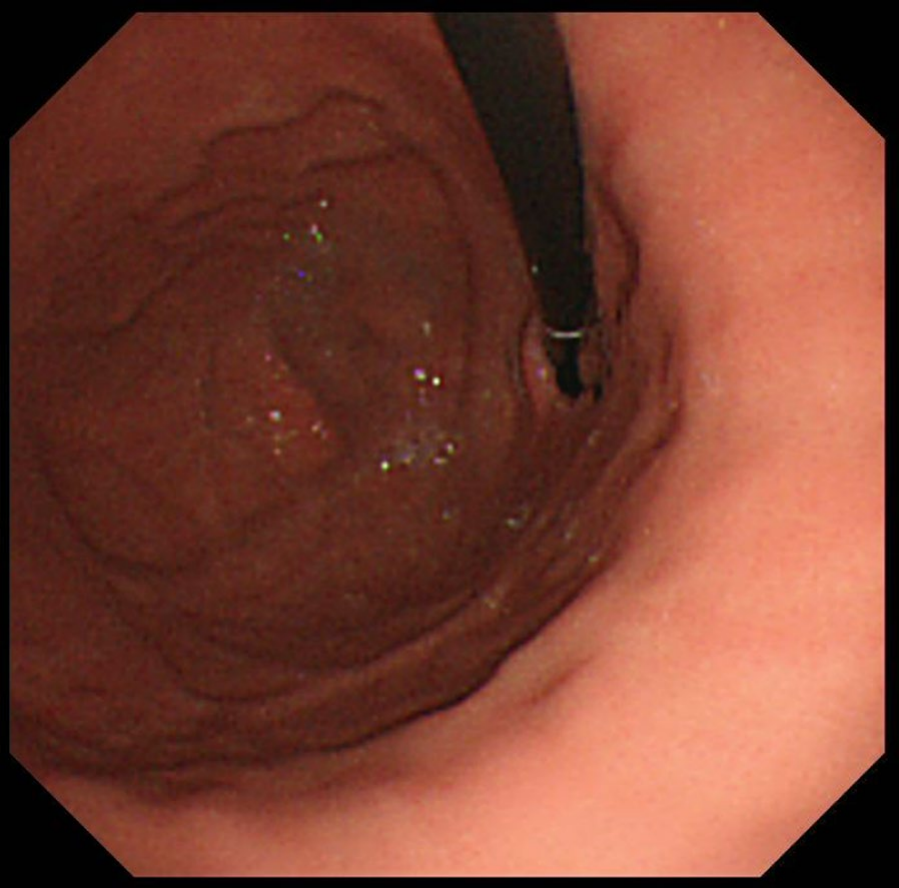
Esophagoduodenoscopy. No gastric varices were identified as a source of bleeding

**Fig. 4:**
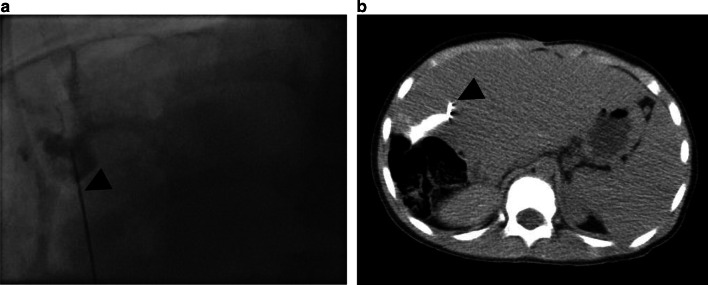
Percutaneous transhepatic puncture and sclerotherapy of the varices was performed. **a** Intraoperative radiograph. **b** Intraoperative computed tomography image. The black arrowhead indicates the needle puncture

In 2020, the patient was diagnosed with severe anemia (Hb 5.6 g/dl) with normal liver function. Although she was not aware of any massive melena, a fecal occult blood test indicated gastrointestinal bleeding. She had no gross or microscopic hematuria. Computed tomography (CT) revealed RY jejunal varices as a source of hemorrhage (Fig. 2a). Gastric varices were also identified (Fig. 2a), which were ruled out as the source of bleeding using esophagogastroduodenoscopy (Fig. 3). The compression of the LRV between the abdominal aorta and supramesenteric artery (SMA) was observed (Fig. 2b, c), which would cause portal hypertension through SRS and pelvic venous congestion through the gonadal vein (Fig. 2d). The angle between the abdominal aorta and the SMA was 27.6° (Fig. 2c), which is small enough for to be used as a diagnostic parameter of Nutcracker syndrome (NCS) [4]. Doppler ultrasonography showed patent RPA and maintained intrahepatic portal vein flow. RY varices were fed from the supramesenteric vein (SMV) (Fig. 2d) and had no direct connection to the RPA. She underwent percutaneous transhepatic RY jejunal variceal sclerotherapy under general anesthesia. Under interventional radiology—CT/angio system, percutaneous transhepatic puncture of the varix of the RY jejunum was performed using a 21-gauge needle using with sonographic guidance and CT guidance. The varices were confirmed by angiography and sclerosed by the injection of 5% ethanolamine oleate iopamidol (Oldamin; Mochida Pharmaceutical, Tokyo, Japan) (Fig. 4). At the same time, liver needle biopsy showed an almost normal liver status without signs of rejection or fibrosis. After sclerotherapy, she was in good condition with no recurrence of anemia for 6 months.

This study was approved by the Tohoku University Research Ethics Committee (2018-1-442). The individual informed consent paperwork was recorded. In preparation for this manuscript, all efforts have been made to protect patient privacy.

## Discussion

In the patient after LT with RPA, variceal bleeding due to thrombosis of RPA was reported as a long-term complication [5]. However, this is the first report of gastrointestinal bleeding from the RY jejunum that is related to compression of the LRV related to RPA in the follow-up period. The compression of the LRV between the abdominal aorta and SMA was first described by De Shepper in 1972 [6] as a rare vascular condition, which is known as a cause of NCS when it results in venous hypertension and symptoms such as hematuria, proteinuria, flank pain, and pelvic venous congestion. The beak sign and the narrow angle between the abdominal aorta and SMA are known as typical CT findings and are useful for diagnosing NCS [4]. These signs were observed in our case, although not immediately after the LT. During follow-up period, Doppler ultrasonography has recorded consistently steady flow of intrahepatic portal vein, with a velocity of about 10 cm H_2_O. It was difficult to identify splanchnic venous hypertension related with the LRV compression in the RPA patient using only ultrasonography of the liver graft. Chu et al. reported that portal hypertension after LT is related to gastrointestinal bleeding from ectopic RY varices in children [7]. The increased venous pressure caused by compression of the LRV in our case seemed to result in RY varices. Liver graft fibrosis could also cause venous pressure, but neither rejection nor fibrosis was confirmed by liver biopsy in our case.

The treatment options for LRV compression include conservative methods, surgical methods [8] and endovascular stenting [9, 10] according to the severity of the symptoms. However, in patients with RPA, management of the compression of the LRV is more complicated because compression of the LRV could affect both splanchnic vein hypertension through the splenorenal shunt and intrahepatic portal vein flow through RPA. In addition, an endovascular approach to the LRV requires the transhepatic route using a hepatic puncture because using the endovascular approach via the IVC is impossible. Surgical transposition of the RPA from the LRV to the SMV could be a curative option. However, we did not adopt it owing to the high risk of insufficient intrahepatic PV flow and graft loss. For younger patients with NCS without severe symptoms, conservative management is reported to be acceptable because symptom resolution would be expected as they grow up [11]. In patients with RPA, conservative management might be preferable if the graft function is maintained and the symptoms related to venous hypertension are controlled.

The treatment of jejunal varices formed at the site of the RY jejunum after liver transplantation has not been established. Endoscopic treatment of RY varices has been reported to be effective [7], and recently, double-balloon enteroscopy for pediatric patients has proven safe and useful [12]. We did not adopt an endoscopic approach because it is difficult to approach the RY jejunum in patients under 10 years of age, even in a high volume center [12]. Transileocolic vein obliteration using interventional radiology was not performed because of the need for laparotomy to expose the ileocolic vein. Stenting to the LRV via the transhepatic route was not performed because the intrahepatic PV flow was maintained at approximately 10 cm H_2_O on Doppler ultrasonography, the stenting required hepatic puncture, and the stenting to the LRV could not directly affect the varices. We opted for percutaneous transhepatic sclerotherapy (PTS), which is less invasive. In the present case, although the sclerosed varices were not confirmed by the follow-up CT, PTS would have an effect on the hemostasis from the varices because repetitive anemia disappeared after the PTS.

## Conclusions

In conclusion, this is the first reported case of bleeding from RY varices caused by compression of the LRV after LDLT with RPA. Portal vein flow after RPA depends on the nonphysiological collateral flow. We think RPA should be cautiously performed for pediatric patients because it is difficult to predict whether the collateral status will be maintained without formation of the varix, especially in pediatric patients who will grow up. If RPA is inevitably performed, long-term follow-up with CT is required to detect venous hypertension and for new collateral formation.

## Data Availability

Not applicable.

## Abbreviations

CT: Computed tomography
IVC: Inferior vena cava
LT: Liver transplantation
LDLT: Living donor liver transplantation
LRV: Left renal vein
NCS: Nutcracker syndrome
PTS: Percutaneous transhepatic sclerotherapy
PV: Portal vein
PVT: Portal vein thrombosis
RPA: Renoportal anastomosis
RY: Roux-en-Y
SMA: Supramesenteric artery
SMV: Supramesenteric vein
SRS: Splenorenal shunt
